# A health professions education editors’ open letter to our community

**DOI:** 10.1007/s40037-018-0444-7

**Published:** 2018-07-19

**Authors:** David P. Sklar, Peter G. M. de Jong, Erik W. Driessen, Kevin W. Eva, Ronald M. Harden, Grace C. Huang, Gail M. Sullivan

**Affiliations:** 1 Academic Medicine, Albuquerque, USA; 2 Medical Science Educator, Leiden, The Netherlands; 3 Perspectives on Medical Education, Maastricht, The Netherlands; 4 Medical Education, Vancouver, Canada; 5 Medical Teacher, Dundee, UK; 6 MedEdPORTAL, Boston, USA; 7 Journal of Graduate Medical Education, Farmington, USA

As editors of journals concerned with health professions education, we take very seriously the influence of our published articles on curriculum development, learner assessment and, ultimately, the quality of health care provided to our communities. As members of the International Editors Group of Health Professions Education Journals, we routinely gather at international meetings to share new ideas, voice concerns and provide support to each other as we grapple with financial, logistical, technical and political challenges faced by our journals. It is therefore appropriate that we clearly communicate our concerns about increasing risks related to current regulations and attitudes affecting international travel, global meetings and global health.

As a community of scholars we must reach out to each other, challenge ideas, and identify the most effective and creative approaches regarding how healthcare education can facilitate better health. Doing so benefits all of us, rich or poor, regardless of race, ethnicity, and language, country of residence or cultural values. Disease knows no borders. While health systems, medical knowledge, skills, and medications evolve in response to our unique local conditions, we never know when something in one of our countries will become important (maybe even life-saving) in another. Healthcare education needs to monitor these global developments closely to warrant the best possible education for our students and practitioners and the best possible healthcare for society. As we determine how to train the future healthcare professionals and leaders, it is imperative that we recognize the benefits of global health education and travel to learn from each other.

We recognize that there are risks from those who consider using travel and international meetings to provoke violence and sow the seeds of international fear and distrust. However, we believe that our greatest counter weapon is our academic community. Key collaborations and mutual understanding have been built by sharing knowledge, keeping eyes and ears open to new evidence, and commitment to peace, health and learning. The trust we have built over many years working together at international meetings is a model that enhances, rather than undermines, global security.

We urge our governments, elected officials, community leaders and health professions leaders to appreciate the value that global meetings can bring to each of our countries. We ask you to provide the freedom for members to participate fully in international meetings and exchanges, regardless of the country from which we come. As we listen and learn from each other and share what we learn in our journals, we help to make the world a healthier and safer place. We ask all of you who are our readers and members of our community of scholars to reach out to your elected officials to ensure they understand how they can, and why they should support our continued international health educational activities and collaborations.

